# The Influence of Different Light Day Distribution in Hy-Line W36 Laying Hens on Egg Production and Egg Quality

**DOI:** 10.3390/ani15060838

**Published:** 2025-03-14

**Authors:** Alexis J. Clark, Ari J. Bragg, Abdulmohsen Hussen Alqhtani, Mireille Arguelles-Ramos, Ahmed Ali

**Affiliations:** 1 Department of Animal and Veterinary Sciences, Clemson University, Clemson, SC 29634, USA; ajclrk@g.clemson.edu (A.J.C.); ajbragg@clemson.edu (A.J.B.); marguel@clemson.edu (M.A.-R.); 2 Department of Animal Production, College of Food and Agriculture Sciences, King Saud University, P.O. Box 2460, Riyadh 11451, Saudi Arabia; ahalqahtani@ksu.edu.sa; 3 Animal Behavior and Management, Department of Veterinary Hygiene, Veterinary Medicine, Cairo University, Cairo 12613, Egypt

**Keywords:** poultry, eggshell quality, feed intake, osteoporosis, artificial light, midnight feeding, scotophase

## Abstract

Laying hens are commonly provided with 16 h of continuous light and 8 h of continuous darkness, with eggshell calcification largely developing during dark hours when dietary calcium is depleted simultaneously with the daily fasting period in laying hens. This phenomenon is thought to be the leading cause of osteoporosis in modern commercial flocks. This trial examined how disrupting the midnight phase affected egg quality in Hy-line W36 hens assigned to control (C) or treatment groups (W1 or W2) from 20 to 70 weeks of age. C hens received 16 h of light and 8 h of dark. W1 hens received 1 h of scotophase interruption, whereas W2 hens received 2 h of scotophase interruption. Across weeks, performance, production, and egg quality measures were measured (*p* < 0.05). Scotophase interruption displayed a positive effect on the treatment groups in terms of HDEP (week 50: *p* = 0.028, 0.016; week 70 = 0.031,0.022), damaged eggs (week 50: *p* = 0.012, 0.021, week 70: *p* = 0.019, 0.025), eggshell weight (week 30: *p* = 0.021, 0.027; week 50: *p* = 0.022, 0.024; week 70; *p* = 0.018, 0.019), eggshell thickness (week 30: *p* = 0.017, 0.022; week 50: *p* = 0.018, 0.031; week 70; *p* = 0.029, 0.033), ash percent week 30: *p* = 0.027, 0.021; week 50: *p* = 0.012, 0.014; week 70; *p* = 0.031, 0.034), and eggshell strength (week 30: *p* = 0.025, 0.023; week 50: *p* = 0.019, 0.021; week 70; *p* = 0.029, 0.015) in W1 and W2 birds compared to C birds.

## Simple Summary

Access to dietary calcium during eggshell formation may impact egg quality and laying-hen welfare. Our study aimed to determine the effects of interrupted darkness (scotophase) on egg quality parameters in laying hens. Hens were housed in pens exposed to either the commercial recommendation for light schedules (C; 16 h light/ 8 h darkness), 1 h of interrupted scotophase (W1; 15 h light/ 4 h dark/ 1 h light/ 4 h dark), or 2 h of interrupted scotophase (W2; 14 h light/ 4 h dark/ 2 h light/ 4 h dark) from 20 to 70 weeks of age. C hens exhibited lower daily egg production rates than W1 and W2 hens during peak and late lay phases. Additionally, C hens exhibited higher percentages of damaged eggs when compared to W1 and W2 hens in the mid- and late-lay phases, as well as lower eggshell weights, thicknesses, eggshell ash percentages, and eggshell strengths. An age effect was detected in measures of external quality for C hens in terms of shell thickness, ash percentages, and eggshell strength, whereas these parameters remained consistent for W1 and W2 hens across the weeks of the trial. An age effect was also detected in hen-day egg production for C, W1, and W2 hens.

## 1. Introduction

The world population is expected to increase intensely over the next 40 years, leading to higher food security directives [1]. Eggs from laying hens are one of the most affordable protein sources available to consumers, leading to genetic selection for increased production, persistence in lay, and sustainability for egg quality [2]. In the commercial industry, deterioration of shell quality and a decrease in egg production are the current parameters for replacing flocks around 72 weeks of age [2]. While researchers continue to study the control mechanisms of egg production and persistence of production, an underlying issue of discussion is that increased production and longer lay cycles are directly associated with the health of the birds.

In an effort to maintain optimal bird health and production, lighting systems are widely employed in intensive management systems in the poultry industry. Intensive management systems used for the rearing of laying hens and egg production allow for modifications of artificial light regimens to maintain constant egg production following sexual maturity as compared to extensive management systems seen on small farms with natural lighting [3]. Artificial light influences physiological processes in laying hens, such as the stimulation of hormone release, internal organ functionality, and metabolic processes that encourage the facilitation of digestion. These interconnected systems influence rates of feed efficiency and egg production in laying hens, whereas natural light allows for seasonality and variability of egg production and quality [4,5,6,7].

Over an approximate 24–26 h physiological process, egg formation occurs in the modern laying hen. Of these 24–26 h, eggshell formation takes approximately 20 h, generally during the nighttime (scotophase) period when feed consumption is ceased [8,9]. Once feed consumption is halted, the body becomes reliant on the calcium within the feed previously collected in the gastrointestinal tract (GIT) to satisfy an elevated calcium requirement during shell calcification [10,11,12]. After roughly 4 h of scotophase, the feed in the GIT is significantly reduced, causing an absence of total dietary calcium accessible for absorption between 5 and 8 h of scotophase [13,14]. In turn, the bird cannot conserve an adequate level of total calcium in the blood for the continuance of eggshell calcification, compelling the body to employ alternative sources of calcium during shell development, predominantly through skeletal stores [13,14,15]. The growing human population and subsequent heightened demand for eggs in recent years has been mediated through selective breeding and improved housing environments for continual production in laying hens. Thus, modern hens lay approximately one egg per day. This stimulates the gradual resorption of medullary and structural bone concurrently due to amplified osteoclastic cell activity [16,17]. Osteoclastic activity of skeletal stores occurs as a biological source of calcium for the formation of the eggshell, causing a rapid depletion of calcium reserves within the body [18]. The progression of bone resorption transpires daily during the scotophase period with little opportunity for structural bone restoration. This is associated with terminated structural bone osteoblastic activity throughout the reproductive period in laying hens, compelling the bird into a state where bone resorption surpasses bone formation [19]. This mechanism is alleged to cause osteoporosis and inferior eggshell quality in laying hens with a habitual negative calcium balance in combination with a prolonged period of high egg production [15,20].

Due to elevated demands for calcium during the lay cycle, the commercial egg industry suffers from high rates of fragile eggshells and bone fractures starting at the beginning of the production phase (early lay) and increases during the late-lay phase. A decrease in blood calcium levels during the scotophase has been associated with the rise in inferior eggshell quality. The characteristics of the eggshell are greatly influenced by genetics, age, environment, and nutrition [21,22]. Components of the eggshell consist of approximately 94% CaCO_3_, 1% MgCO_3_, 1% Ca_3_(PO_4_)_2_, and 4% organic compounds, where the calcium content motivates shell fragility and brittleness, while phosphorus motivates resiliency and elasticity [23,24]. Previous publications have explored the deterioration of eggshell quality over the span of each lay phase, finding increases in deterioration with increases in age [2,25,26]. Producers see an average loss of revenue, reaching approximately 8% early in the lay phase and extending up to 20–30% later in the production phase (65–70 weeks of age) [27]. Inferior eggshell quality is of concern for consumer safety, as damaged eggshells increase the chance of interior contamination with bacteria [28].

Calcium is a macro-element critically required for physiological needs in laying hens. Calcium is especially important in regard to the bone health and eggshell calcification process during egg formation. Previous studies have investigated various particle sizes, calcium sources and increases in calcium percentages within the diet [8,10,11,29]. However, some studies have indicated that increasing calcium within the diet, although the most obvious, is not a feasible solution to depressing the rate of osteoporosis, as the capacity for calcium absorption cannot be increased greater than what is currently used in standard commercial diets [8,30]. Additionally, the studies investigating particle size have found solutions indicative of maintaining calcium for a longer period in the GIT during the scotophase. Conversely, these studies still show that inadequate dietary calcium levels are met late during the scotophase. Furthermore, other studies investigated the effect of increasing light hours available to the birds (photophase) to allow them to consume feed for a longer period. Although this strategy developed better egg quality in a commercial setting, allotting a longer photophase period would allow for higher feed intake, ultimately causing a rise in production costs and having the potential to inhibit bird welfare [31].

Therefore, a new approach is necessary for the improvement of hen welfare and production without the inflation of feed costs. A promising intervention is to interrupt the scotophase period with 1 or 2 h of continuous light while simultaneously reducing the photophase period by the same amount of time. This will allow the birds to replenish dietary calcium in the GIT during eggshell calcification without increasing the total hours of light per day or feed intake. We hypothesized that birds given 1 or 2 h of light during the midnight period would have increased blood calcium levels and would produce better eggshell quality without impacting egg-laying production and the overall performance of laying hens. In turn, our objective was to examine the effects of scotophase interruption on eggshell quality, egg production, and hen performance parameters without increasing the total amount of daily light hours.

## 2. Materials and Methods

### 2.1. Ethics

This research was accepted by the Clemson University Institutional Animal Care and Use Committee (protocol # AUP2020-0050) and followed all IACUC guidelines for animal use.

### 2.2. Animals and Husbandry

This study was conducted at the Morgan Poultry Center at Clemson University in Clemson, South Carolina, USA. Seventeen-week-old Hy-Line W36 chicks (*n* = 396) were randomly allocated into 18-floor pens (6 pens/treatment; 22 birds/pen) and equipped with a unique identification number via neck tag, as seen in the corresponding study [32]. The trial took place from 20 to 70 weeks of age, succeeding a three-week acclamation period. After the acclimation period, the birds were assigned and exposed to three different lighting schedules dependent on their randomly allocated pens (Control (C), Treatment Group 1 (W1), and Treatment Group 2 (W2)). The C birds were given the commercial standard lighting schedule (16 h light: 8 h dark), whereas the treatment groups were given either one or two hours of interrupted scotophase (W1 and W2, respectively). The intensity of the lighting program was set to 30 Lux with an LED light spectrum of combined 3000 K white and 630 nm red wavelengths. The domes and light programs were designed through NatureDynamics (ONCE by Signify, Plymouth, MN, United States) with a corresponding Interact Agriculture platform (Signify Netherlands B.V., The Netherlands).

Identical experimental floor pens (4.3 m^2^) were used and bedded with 5 cm of clean wood shavings. The poultry house used in the trial was fully enclosed and climate-controlled, preventing the impediment of temperature from outside sources. Each pen contained 10 nest boxes (0.2 m^2^/ nest box) and wooden perches (15 cm perch space/bird). The pens were distributed across the house and tailored on the fronts and sides with thick, black, heavy-duty curtains in order to eliminate light interference across treatments without impeding ventilation. Proper ventilation was achieved using tunnel fans and side-wall inlets. The use of cool-cell pads along with fans helped ensure favorable temperatures were kept in the summer, while industrial heaters, which were used to ensure favorable temperatures, were kept during the winter. The birds were given access to feed and water ad libitum. All birds across treatments were fed the same commercially prepared diet after a prerequisite phase-fed formulation from 1 to 20 weeks of age (Table 1).

### 2.3. Measures of Performance

During weeks 20, 30, 50, and 70, all eggs were collected in each pen at two time points each day over a 3-day period. All eggs collected were identified and weighed on an electronic scale. Broken, cracked, and soft eggs were taken into account. Broken eggs were classified as any egg with identifiable damage to the outer shell that would be deemed “uncollectable”. Such damage could occur in the form of holes or cracks. Eggs that were consumed by the birds between collection times (as designated with declining egg counts) were also recorded as damaged. During the same 3-day period, feed offered and refused was recorded and used to calculate feed conversion per egg weight (kg/kg) and the average daily feed intake per bird in each treatment [33]. The following formula was utilized for the analysis of the feed conversion ratio (FCR):

$$ADFI=\frac{Feed\;Offered-Feed\;Refused}{\#\;Days*\#\;Birds}\;\;\;\;\;\;\;\;FCR=\frac{ADFI}{Egg\;Weight}$$

### 2.4. Measures of Egg Quality

#### 2.4.1. Collection and Evaluation

From the total eggs collected, 60 eggs were selected per treatment (*n* = 180 eggs/week) through a candling process for further assessment of quality and characteristics (avoiding broken, cracked, or dirty eggs). Following candling, these eggs were measured for length and width using a manual caliper (PetCure Oncology).

#### 2.4.2. Breaking Strength and Internal Quality Parameters

Following the length and width analysis of each egg, breaking strength was recorded using an Egg Force Reader (EA27432021; Okra Food Technology, Ltd., West Bountiful, UT, USA). A single egg was placed on a cradle under the force reader arm. The force reader exerted vertical pressure until it experienced negative force via a crack or break of the eggshell. Following breaking strength analysis, the eggs were cracked onto a glass surface for the assessment of internal egg quality parameters, such as albumen height, yolk weight, yolk color, and Haugh unit. The albumen height was measured using a Digital Haugh Tester (Baxlo Precision; Polinyà, Barcelona, Spain). The yolk weight was measured (g) following the separation of the yolk from the albumen using a straining technique, ensuring the prevention of breaking the yolk. The yolk color was measured following separation using a yolk color fan (DSM YolkFan, OKRA Food Technology LLC, West Bountiful, UT, USA). The Haugh unit was calculated following the conduction of internal analysis using the equation HU = 100 × log(h − 1.7 × w^0.37^ + 7.57), where “h” is the albumen height in millimeters, and w is the weight of the egg in grams. Following internal egg quality assessment, the eggshells were washed in deionized water and placed to dry for further analysis.

#### 2.4.3. External Egg Quality Analysis

Following a 48 h drying period, the eggshells were weighed, and eggshell thickness was analyzed using a digital caliper with 0.01 mm precision. Three points of interest were measured within the egg’s equatorial region and averaged for eggshell thickness measurements.

### 2.5. Eggshell Ash Percentage

The eggshells collected from weeks 20, 30, 50, and 70 (*n* = 180/week) were analyzed for mineral content through an ashing process. Following internal and external quality parameter measurements, the eggshells were broken into small pieces, placed into ceramic crucibles of known weight, and subsequently weighed. The eggshells were placed into an ash oven and ashed for 6 h at 600 °C. The oven utilized in this experiment was the Thermolyne 30400, Barnstead International, IA, USA. Following ashing, the crucible was positioned within a desiccator for a single hour, and the weight of the ash was recorded. To analyze the proportion of eggshell ash, we divided the eggshell ash weight by the eggshell dry weight and multiplied by 100.

### 2.6. Statistical Analysis

The minimal sample size was determined utilizing a power analysis, indicating that our flock was sufficient to show statistical significance for each parameter measured. The data were investigated using the “stats” package (R Core Team, 4.3.2, 2013) in R software (version 3.3.1). Generalized linear mixed models (GLMMs) were applied to evaluate the impact of treatment and bird age on each variable. The “lme4 1.1-36” package was used for the construction of these models [34]. For each GLMM, the interaction between the main effects was also evaluated as a fixed effect, and the pen was characterized as a random effect. The “Quasibinomial” family was utilized for proportion data (i.e., egg production %), and the “Poisson” family was utilized for other data types. Post hoc analyses were performed using Tukey’s Honestly Significant Difference (HSD) test procedures, with a significance level set at α = 0.05 [35]. A “DHARMa 0.4.7” package was used to evaluate model residuals and GLMM assumptions. To evaluate the normality of model residuals (i.e., eggshell strength (N)), a Shapiro–Wilk test was employed. A “Psych 2.4.12” package was employed to compute descriptive statistics and displayed as mean ± standard error mean (SEM).

## 3. Results

### 3.1. Performance Results

#### 3.1.1. Average Daily Feed Intake

**Figure 1:**
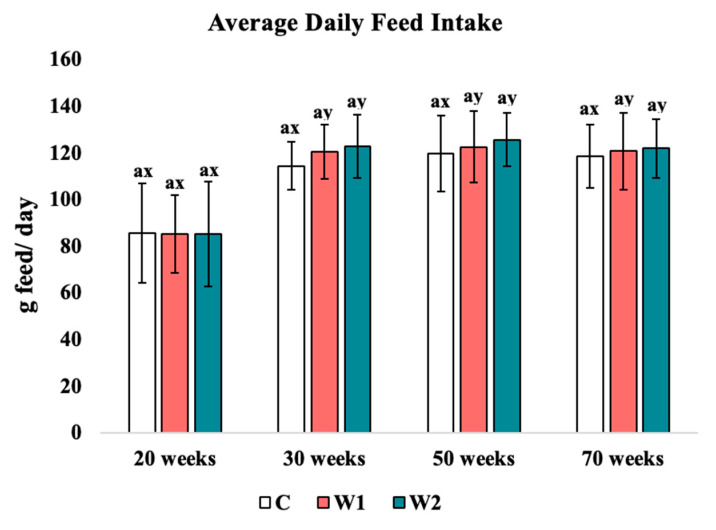
Average daily feed intake: Treatments: C: 16 h light (photophase) with 8 h of continuous darkness (scotophase). W1: 15 h photophase, 4 h of scotophase, 1 h of photophase, then 4 h of scotophase; W2: 14 h photophase, 4 h of scotophase, 2 h of photophase, then 4 h of scotophase. ^a^ Means with differing superscripts indicate statistically significant differences across treatment within the same week at *p* < 0.05. ^x,y^ Means with differing superscripts indicate statistically significant differences within the same treatment across weeks at *p* < 0.05.

Treatment had no significant effect on the average daily feed intake (ADFI; Figure 1) across all weeks of the study. While no significant effects were detected in ADFI in C hens, age had a significant effect on ADFI (*p* = 0.034) at week 20 compared to 30, 50, and 70 of age for W1 and W2 birds (W1: *p* = 0.023, 0.032, 0.035, W2: *p* = 0.033, 0.031, 0.030, respectively).

#### 3.1.2. Feed Conversion Ratio

**Figure 2:**
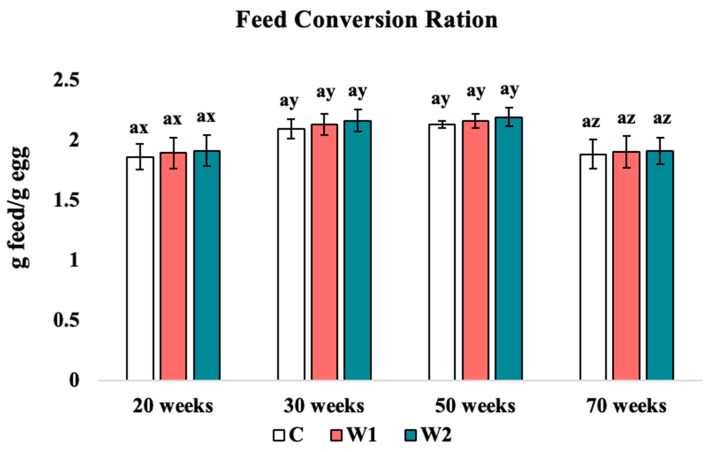
Feed conversion ratio: Treatments: C: 16 h light (photophase) with 8 h of continuous darkness (scotophase). W1: 15 h photophase, 4 h of scotophase, 1 h of photophase, then 4 h of scotophase; W2: 14 h photophase, 4 h of scotophase, 2 h of photophase, then 4 h of scotophase. ^a^ Means with differing superscripts indicate statistically significant differences across treatment within the same week at *p* < 0.05. ^x,y,z^ Means with differing superscripts indicate statistically significant differences within the same treatment across weeks at *p* < 0.05.

No significant change in the feed conversion ratio (FCR) was found across treatments within each week of data collection. However, a significant effect of age on the FCR was detected across weeks (*p* = 0.022; Figure 2). Week 70 showed the lowest FCR compared to week 20, 30, and 50 (C: *p* = 0.021, 0.028, 0.033, W1: *p* = 0.026, 0.032, 0.034, W2: *p* = 0.029, 0.032, 0.034, respectively); week 20 was significantly lower when compared to weeks 30 and 50 (C: *p* = 0.029, 0.025, W1: *p* = 0.023, 0.031, W2: *p* = 0.024, 0.034, respectively), whereas the FCRs for all treatment groups were not significantly different at 30 and 50 weeks of age.

#### 3.1.3. Hen/Day Egg Production

**Figure 3:**
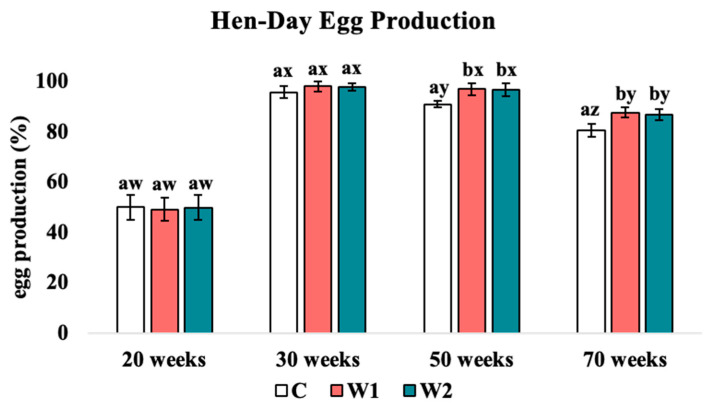
Hen/day egg production: Treatments: C: 16 h light (photophase) with 8 h of continuous darkness (scotophase). W1: 15 h photophase, 4 h of scotophase, 1 h of photophase, then 4 h of scotophase; W2: 14 h photophase, 4 h of scotophase, 2 h of photophase, then 4 h of scotophase. ^a,b^ Means with differing superscripts indicate statistically significant differences across treatment within the same week at *p* < 0.05. ^w,x,y,z^ Means with differing superscripts indicate statistically significant differences within the same treatment across weeks at *p* < 0.05.

An effect of treatment on hen/day egg production (HDEP) was detected in the late-lay phase (*p* = 0.023, Figure 3), where C hens produced the lowest HDEP when compared to W1 (week 50: *p* = 0.028, week 70; *p* = 0.031) and W2 (week 50: *p* = 0.016, week 70; *p* = 0.022) birds. No significant differences were detected between W1 and W2 treatments. Additionally, age significantly affected hen daily egg production (HDEP; *p* = 0.021). Week 20 showed the lowest HDEP compared to 30, 50, and 70 weeks of age (C: *p* = 0.019, 0.022, 0.028, W1: *p* = 0.018, 0.021, 0.024, W2: *p* = 0.027, 0.025, 0.031, respectively), while the HDEP at week 70 was significantly lower than 30 and 50 weeks of age (C: *p* = 0.015, 0.021, W1: *p* = 0.026, 0.029, W2: *p* = 0.017, 0.023, respectively).

### 3.2. Egg Quality Results

#### 3.2.1. Internal Egg Quality

The effect of treatment on the internal egg quality was investigated (Table 2). Significant effects of treatment were detected in the percentage of damaged eggs at weeks 50 and 70 (*p* = 0.021). During these weeks, C hens laid a significantly higher percentage of damaged eggs than W1 and W2 (week 50: *p* = 0.012, 0.021, week 70: *p* = 0.019, 0.025, respectively). There were no significant effects of treatment detected for the egg weight, albumen weight, yolk weight, yolk color, or Haugh Unit across weeks 20–70. However, a significant effect of age was found for the egg weight, albumen weight, and damaged eggs. For all treatment groups, a significant difference in the egg weight was detected between weeks 20 compared to all the other weeks of the study (*p* = 0.027), while weeks 30, 50, and 70 were not significantly different. Similarly, albumen weights at 20 were significantly lower compared to the other weeks (*p* = 0.026), while weeks 30, 50, and 70 were not significantly different. Significant differences were detected at weeks 30 and 50 for the ratio of damaged eggs in C hens (*p* = 0.017, 0.022, respectively), while weeks 20 and 30 were not significantly different, and weeks 50 and 70 were not significantly different. No significant differences in the ratio of damaged eggs were detected across weeks for W1 or W2 hens. No significant effect of age was detected for the yolk weight, yolk color, or Haugh unit in this study.

#### 3.2.2. External Egg Quality

The effect of age and treatment were investigated for external egg quality parameters to investigate the eggshell weight, thickness, ash percentage, and strength (Table 3). A significant effect of treatment was detected in all external parameters at weeks 30, 50, and 70. The eggshell weight for C hens was significantly lower than W1 and W2 hens at each week following week 20 (week 30: *p* = 0.021, 0.027; week 50: *p* = 0.022, 0.024; week 70; *p* = 0.018, 0.019). The eggshell thickness showed similar results, where eggs from the C treatment had thinner shells after week 20 when compared to W1 and W2 treatments (week 30: *p* = 0.017, 0.022; week 50: *p* = 0.018, 0.031; week 70; *p* = 0.029, 0.033). Eggshell ashing percentages (week 30: *p* = 0.027, 0.021; week 50: *p* = 0.012, 0.014; week 70; *p* = 0.031, 0.034) and eggshell strength (week 30: *p* = 0.025, 0.023; week 50: *p* = 0.019, 0.021; week 70; *p* = 0.029, 0.015) results also showed that C hens exhibit less mineral deposition and weaker eggshells than W1 and W2 hens after week 20.

Additionally, the effect of age was detected across treatments for most parameters. No significant differences in eggshell weight were detected in C hens across weeks, while W1 and W2 hens showed the lowest eggshell weight at week 20 compared to 30, 50, and 70 weeks of age (W1: *p* = 0.023, 0.021, 0.034; W2: *p* = 0.021, 0.032, 0.033, respectively). However, eggshell weights across weeks 30, 50, and 70 were not significantly different. The effect of age on eggshell thickness was detected at weeks 30, 50, and 70, where C hens showed the highest eggshell thickness at week 20 compared to 30, 50, and 70 weeks of age (*p* = 0.025, 0.026, 0.033, respectively), while W1 and W2 showed no significant differences in eggshell thickness across weeks. Similarly, significant differences in age on eggshell ash% and strength in C hens were detected, where C hens showed the highest ash% and eggshell strength at week 20 compared to 30, 50, and 70 weeks of age (ash%: *p* = 0.012, 0.016, 0.021; eggshell strength: *p* = 0.023, 0.026, 0.031, respectively), while weeks 30, 50, and 70 were not significantly different.

## 4. Discussion

### 4.1. Average Daily Feed Intake and Feed Conversion Ratio

Average daily feed intake in laying hens is expected to increase with age and fluctuate with the lay phase [2]. Previous studies have focused on various colors of monochromatic lights and their effects on performance data. These studies have indicated that increasing age is associated with the reduced ability to absorb calcium in the GIT, causing the incidence of osteoporosis in laying hens [36,37,38]. Some intermittent lighting regimes have been investigated and shown to improve FCR [39,40]. Other studies have indicated that intermittent light regimens allow for a decreased consumption rate due to the darkness period during the photophase, thus improving FCR [41]. A peak in feed consumption was stimulated by the onset of a “dusk” period, where the hens consume feed to fill their crops to prevent food deficiencies during the scotophase period in previous studies [42,43]. The timing of calcium intake and variation in calcium supply within the diet has been studied in previous experiments, where it was concluded that hens could not maintain optimal shell quality when they consumed approximately 2- or 3-day supply of calcium in only 1 day [37]. In the current study, age had a significant effect on W1 and W2 hens when compared to C hens. This can likely be associated with natural feeding behaviors or with the increased amount of “dusk” periods where increased feed consumption is stimulated before the scotophase. It is relevant to think that the increase in ADFI could be associated with other egg quality factors due to the deposition of calcium onto the eggshell. These findings could align with previous findings of increased feed consumption prior to the scotophase; however, more research on feeding behavior is needed [42,43]. Studies that utilized the same genetic line of Hy-line W36 hens observed similar results for ADFI and FCR, nearing 125 g of feed intake and 2.18 g of feed per gram of egg produced [44]. An effect of age was also found in the feed conversion ratio of all hens in the current study, in alignment with the stage of lay phase. C, W1, and W2 hens demonstrated significantly higher feed conversion ratios from 20 to 30 weeks of age and a significantly lower FCR from 50 to 70 weeks. A decrease in the absorption of calcium has been associated with the continuation of the lay cycle and age of laying hens, aligning with the results of the current study.

### 4.2. Hen Daily Egg Production

Previous studies have indicated an effect of lighting regimens on egg production in laying hens [7,39,45]. Artificial lighting interventions are an important husbandry tool in the laying-hen industry. Studies have indicated an increase in egg production among hens exposed to intermittent lighting schedules in comparison to hens exposed to the commercial recommendation of 16 h of continuous photophase and 8 h of scotophase. Some studies presented have found significant influences of light regimens on egg production, with an approximate 5.60% increase [46,47,48,49,50,51]. It is also stated that hens receiving a longer photophase experience higher production rates compared to hens given short photoperiods [51]. Other studies have found contradictory findings of lower production rates with intermittent light schedules [52]. The current study found that age and treatment had significant effects on HDEP, where C hens showed the most variation. C, W1, and W2 hens should have similar changes from early to peak lay phases (weeks 20 and 30), where HDEP increased significantly during peak lay. Following week 30, C hens began to significantly decline in HDEP at week 50, while W1 and W2 hens remained constant. At 70 weeks of age, a significant decline in HDEP was found in C, W1, and W2 hens compared to 50 weeks of age. Similarly, C hens showed significantly lower HDEP than W1 and W2 hens. In a study using Hy-line W36 hens, researchers found similar results for daily egg production at week 50, reaching slightly above 82% production, exhibiting normalcy among genetic strains [44]. This aligns with the natural decrease in HDEP with the continuation of the lay phase, as well as findings reporting that intermittent lighting programs result in an increase in production rates [40,46,47,48,49,50]. This can likely be attributed to the physiological mechanisms related to the interruption of scotophase, including the reduction of calcium resorption from skeletal stores, thus improving welfare through the reduction in bone fractures and associated pain [15].

### 4.3. Internal Egg Quality

In our study, we did not hypothesize interventional changes in internal egg quality due to the lack of nutritional changes; however, we did expect natural changes in internal quality in relation to age. Normal changes in internal egg quality have been shown in previous studies in relation to the stage of the lay phase the hen is in [27]. Higher egg weights have been associated with increasing age of laying hens, while egg quality tends to deteriorate [2]. Previous studies involving intermittent lighting schedules found increases in egg weights [52], while other reports found consistency in egg weights [53]. The current study demonstrated changes in egg and albumen weights in relation to age. Subsequently, no changes in egg weights were found across treatments when compared to the control hens. The Haugh unit is a standard measure of albumen quality in the industry. A decline in Haugh units has been associated with the increase in hen age. A previous study indicated that the average Haugh unit decreased from 89.6 to 68.8 as the hens aged [54]. Other studies have indicated that egg size and Haugh unit were not affected by varying light regimes [55]. Additional studies indicate that hens kept under continuous light had higher egg quality traits than hens exposed to intermittent light regimens [39]. The current study demonstrated no changes in Haugh units in relation to age or treatment, contrasting with previous findings of changes in relation to age. A previous study provided similar results for Haugh Unit and yolk color among Hy-line W36 hens [44]. Additionally, the percentages of cracked or deformed eggshells and broken eggs have increased with age, according to previous studies [2,56]. In the current study, an increase in damaged eggs was reported in C hens in relation to age and treatment. W1 and W2 hens had significantly lower rates of broken eggs at weeks 50 and 70 when compared to C hens. This could be attributed to the interruption of scotophase, where treatment hens were allotted a period for dietary calcium replenishment during times of high calcium demand compared to C hens exposed to continuous darkness during eggshell formation. Changes in yolk weight and yolk color are commonly associated with nutritional changes. The current study allowed all birds to consume the same feed over the duration of the experiment, and no changes in yolk color or yolk weight were recorded.

### 4.4. External Egg Quality

The eggshell contains approximately 94% of CaCO_3_, 1% of MgCO_3_, 1% of Ca_3_(PO_4_)_2_, and 4% organic substances consistent with albuminous character [2,57]. This equates to a calcium content of between 28 and 41% and a phosphorus content of approximately 0.102% in the eggshell [23]. The amount of calcium within the eggshell is relative to fragility and brittleness, while phosphorus is relative to elasticity [23]. External characteristics of egg quality are also influenced by many factors, including genetics, age, nutrition, and environment [23,27]. The shell is composed, in part, of calcite columnar crystals that form from the mammillary tissue and are anchored onto the shell membranes. The brittle calcite crystals are supplemented with organic materials that aid in support, creating the tough material visual on the outside of an egg [27]. Eggshell strength is vital to the integrity and safety of egg contents for human consumption [58]. The internal components of the egg are protected from involuntary impacts, microbial pollution, and dehydration by the eggshell. Increases in production have been associated with decreased egg quality parameters, such as eggshell thickness and strength [59]. Interestingly, one group of researchers found that eggshell thickness was reduced linearly with every hour of increased photoperiod, while others reported that eggshell thickness was not differentiated with light regimes [39,46,60]. Eggshell thickness in the current study was affected by treatment and age, where C hens were significantly thinner compared to W1 and W2 hens after 20 weeks of age. Age effects on eggshell thickness were demonstrated in C hens between 20 and 30 weeks of age and, although not significantly different, numerically declined between 30 and 70 weeks of age. The eggshell thickness in W1 and W2 hens remained constant across all weeks and can likely be associated with the interruption of the scotophase and similar rates of calcium absorption in the GIT. These findings align with previous findings, indicating that age, increased production, and increased photophase affect the eggshell thickness [23,27,46,59]. The current study found that the eggshell weight was affected by treatment but not by age. C hens had a significantly lower eggshell weight than W1 and W2 hens starting at 30 weeks. This can likely be attributed to the interruption of the scotophase period, where dietary calcium was restored and readily available for the calcification of the eggshells. Mineralization of the eggshell is associated with the ash content, exhibited following an ashing process. Here, increased calcium contents are indicated by higher ash remnants. In the current study, treatment and age had a significant effect on ash percentage. C hens showed a significantly lower ash percentage at weeks 30, 50, and 70 compared to W1 and W2 hens. C hens also showed progressively lower ash percentages over weeks 50 and 70, aligning with the natural deterioration of eggshell quality with lay phase and age [27,59]. W1 and W2 hens remained constant across weeks, likely attributed to similar absorption rates of dietary calcium during eggshell calcification. Eggshell strength is a measure of force withheld by the shell before failure and is indicative of calcium contents within the shell [23,27]. In this study, eggshell strength differed in C hens in comparison to W1 and W2 hens. Age and treatment had a significant impact on eggshell strength in C hens, where lower values were recorded after 20 weeks of age, while eggshell strength in W1 and W2 hens remained constant. These findings are consistent with previous studies, as strength deteriorates with age and continuation of the lay cycle [23,27].

## 5. Conclusions

The results from this study suggest that changes in the common industry light schedules may lead to improved egg production and external egg quality parameters with advanced age. Similarly, the accessibility of dietary calcium during eggshell calcification aligned with improvements in external egg quality parameters. Therefore, interrupted scotophase has positive effects on feed conversion ratios, production rates, and eggshell quality parameters. Further studies on behavior are needed to define the increases in average daily feed intake for the treatment groups as well as welfare parameters.

## Figures and Tables

**Table 1:** Guaranteed analysis of commercially formulated diet.

Guaranteed Analysis	
Crude Protein (Min.)	16%
Lysine (Min.)	0.85%
Methionine (Min.)	0.36%
Crude Fat (Min.)	3%
Crude Fiber (Max.)	6.5%
Calcium (Min.)	4.1%
Calcium (Max.)	4.4%
Phosphorus (Min.)	0.6%
Salt (Min.)	0.45%
Salt (Max.)	0.8%

**Complete Ingredients List:** Wheat middlings, ground corn, calcium carbonate, dehulled soybean meal, deoiled corn distillers grains, grain distillers dried yeast, heat stabilized rice bran, salt, yeast culture (Saccharomyces cervisiae), manganous oxide, zinc oxide, wheat flour, dried Trichoderma longibrachiatum fermentation extract, niacin supplement, dehydrated Pichia pastoris fermentation extract, mineral oil, basic copper chloride, vitamin A supplement, vitamin D3 supplement, calcium pantothenate, menadione sodium bisulfite complex (source of vitamin K activity), riboflavin supplement, sodium selenite, pyridoxine hydrochloride, vitamin B12 supplement, thiamine mononitrate, ethylenediamine dihydroiodide, folic acid, biotin, choline chloride, vitamin E supplement, ferrous sulfate, hydrated sodium calcium aluminosilicate, L-threonine, L-lysine monohydrochloride, dried Lactobacillus acidophilus fermentation product, dried Enterococcus faecium fermentation product, dried Pediococcus acidilactici fermentation product, dried Trichoderma reesei fermentation extract and solubles, dried Macleaya cordata extract, anise, cinnamon, thyme, garlic essential oil, clove bud essential oil, oregano essential oil, ginger essential oil, dried extracted Streptomyces fermentation solubles, Tagetes (Aztec marigold) extract, vegetable oil, monocalcium phosphate, and DL-methionine hydroxy analogue.

**Table 2:** Internal egg quality measurements.

*Parameter*	*Treatment*	*20 Weeks*	*30 Weeks*	*Parameter*	*70 Weeks*
*Egg Weight (g)*	C	45.68 ± 1.89 ^ax^	63.58 ± 2.33 ^ay^	61.52 ± 2.12 ^ay^	61.58 ± 2.55 ^ay^
	W1	45.26 ± 1.52 ^ax^	62.85 ± 1.58 ^ay^	63.09 ± 1.01 ^ay^	61.16 ± 1.78 ^ay^
	W2	44.98 ± 1.32 ^ax^	63.62 ± 2.52 ^ay^	62.58 ± 1.98 ^ay^	62.09 ± 1.85 ^ay^
*Albumen Weight (g)*	C	26.96 ± 0.96 ^ax^	40.93 ± 0.85 ^ay^	39.61 ± 1.01 ^ay^	39.36 ± 0.78 ^ay^
	W1	26.69 ± 1.12 ^ax^	39.97 ± 0.79 ^ay^	40.71 ± 0.88 ^ay^	38.89 ± 0.78 ^ay^
	W2	26.28 ± 1.45 ^ax^	40.67 ± 1.52 ^ay^	40.23 ± 1.02 ^ay^	39.74 ± 1.12 ^ay^
*Yolk Weight (g)*	C	11.89 ± 3.22 ^ax^	14.88 ± 2.52 ^ax^	14.36 ± 2.52 ^ax^	14.96 ± 2.56 ^ax^
	W1	12.01 ± 1.23 ^ax^	14.96 ± 1.85 ^ax^	14.52 ± 1.66 ^ax^	14.58 ± 1.85 ^ax^
	W2	12.23 ± 2.52 ^ax^	14.63 ± 1.59 ^ax^	14.55 ± 2.46 ^ax^	14.96 ± 3.06 ^ax^
*Yolk Color*	C	8.09 ± 2.2x ^ax^	8.96 ± 1.63 ^ax^	8.23 ± 3.03 ^ax^	8.67 ± 2.35 ^ax^
	W1	8.12 ± 1.25 ^ax^	8.86 ± 1.22 ^ax^	8.52 ± 2.85 ^ax^	8.76 ± 1.96 ^ax^
	W2	8.22 ± 1.96 ^ax^	8.63 ± 2.63 ^ax^	8.85 ± 2.55 ^ax^	8.89 ± 3.66 ^ax^
*Haugh Unit (HU)*	C	75.63 ± 1.96 ^ax^	76.89 ± 2.52 ^ax^	78.58 ± 2.03 ^ax^	80.23 ± 2.16 ^ax^
	W1	76.52 ± 1.55 ^ax^	78.25 ± 3.52 ^ax^	76.58 ± 3.52 ^ax^	79.58 ± 2.88 ^ax^
	W2	77.85 ± 2.03 ^ax^	77.25 ± 2.85 ^ax^	78.55 ± 1.69 ^ax^	80.99 ± 2.11 ^ax^
*Damaged Eggs Ratio (%)*	C	1.03 ± 5.23 ^ax^	3.52 ± 1.55 ^ax^	7.88 ± 1.69 ^ay^	11.25 ± 2.69 ^ay^
	W1	2.52 ± 3.23 ^ax^	1.22 ± 1.89 ^ax^	1.99 ± 1.85 ^bx^	1.85 ± 2.96 ^bx^
	W2	1.66 ± 3.52 ^ax^	1.52 ± 1.88 ^ax^	1.58 ± 1.69 ^bx^	2.09 ± 3.59 ^bx^

Treatments: C: 16 h light (photophase) with 8 h of continuous darkness (scotophase). W1: 15 h photophase, 4 h of scotophase, 1 h of photophase, then 4 h of scotophase; W2: 14 h photophase, 4 h of scotophase, 2 h of photophase, then 4 h of scotophase. ^a,b^ Means with opposing superscripts indicate statistical differences across treatment inside the same week at *p* < 0.05. ^x,y^ Means with opposing superscripts indicate significant differences inside the same treatment across weeks at *p* < 0.05.

**Table 3:** External egg quality measurements.

*Parameter*	*Treatment*	*20 Weeks*	*30 Weeks*	*50 Weeks*	*70 Weeks*
*Eggshell Weight (g)*	C	7.16 ± 0.36 ^ax^	7.21 ± 0.13 ^ax^	7.56 ± 0.16 ^ax^	7.57 ± 0.12 ^ax^
	W1	7.09 ± 0.46 ^ax^	7.99 ± 0.14 ^by^	7.89 ± 0.15 ^by^	7.90 ± 0.16 ^by^
	W2	7.12 ± 0.31 ^ax^	7.96 ± 0.15 ^by^	7.92 ± 0.12 ^by^	7.91 ± 0.16 ^by^
*Eggshell thickness (mm)*	C	0.46 ± 0.09 ^ax^	0.38 ± 0.02 ^ay^	0.36 ± 0.03 ^ay^	0.34 ± 0.04 ^ay^
	W1	0.46 ± 0.08 ^ax^	0.43 ± 0.03 ^bx^	0.42 ± 0.02 ^bx^	0.40 ± 0.02 ^bx^
	W2	0.45 ± 0.05 ^ax^	0.44 ± 0.03 ^bx^	0.41 ± 0.03 ^bx^	0.41 ± 0.03 ^bx^
*Eggshell ash%*	C	96.63 ± 0.63 ^ax^	94.69 ± 0.56 ^ay^	94.23 ± 0.26 ^ay^	94.06 ± 0.34 ^ay^
	W1	96.85 ± 0.99 ^ax^	96.62 ± 0.66 ^bx^	96.06 ± 0.36 ^bx^	95.89 ± 0.22 ^bx^
	W2	96.06 ± 0.58 ^ax^	96.36 ± 0.43 ^bx^	95.96 ± 0.39 ^bx^	95.91 ± 0.29 ^bx^
*Eggshell strength (N)*	C	40.36 ± 4.63 ^ax^	26.88 ± 2.56 ^ay^	24.85 ± 3.52 ^ay^	24.25 ± 2.59 ^ay^
	W1	41.12 ± 3.52 ^ax^	36.56 ± 5.63 ^bx^	37.63 ± 6.25 ^bx^	36.12 ± 3.52 ^bx^
	W2	39.85 ± 4.25 ^ax^	37.89 ± 3.52 ^bx^	39.62 ± 5.69 ^bx^	39.69 ± 6.52 ^bx^

Treatments; C: 16 h light (photophase) with 8 h of continuous darkness (scotophase). W1: 15 h photophase, 4 h of scotophase, 1 h of photophase, then 4 h of scotophase; W2: 14 h photophase, 4 h of scotophase, 2 h of photophase, then 4 h of scotophase. ^a,b^ Means with differing superscripts indicate statistically significant differences across treatment within the same week at *p* < 0.05. ^x,y^ Means with differing superscripts indicate statistically significant differences within the same treatment across weeks at *p* < 0.05.

## Data Availability

Data are contained within the article.

## References

[1] Farsund A.A., Daugbjerg C., Langhelle O. (2015). Food security and trade: Reconciling discourses in the Food and Agriculture Organization and the World Trade Organization. Food Secur..

[2] Bain M.M., Nys Y., Dunn I.C. (2016). Increasing persistency in lay and stabilising egg quality in longer laying cycles. What are the challenges?. Br. Poult. Sci..

[3] Różewicz M. (2023). Effect of Age on Egg Quality of Lakenvelder Hens Kept Under Extensive Rearing Conditions. Int. J. Poult. Ornam. Birds Sci. Technol..

[4] Türker İ., Kalebaşi S. (2009). Effect of fluctuate lighting on performance of laying hens (Short Communication). Arch. Anim. Breed..

[5] Ma H., Li B., Xin H., Shi Z., Zhao Y. (2013). Effect of Intermittent Lighting on Production Performance of Laying-Hen Parent Stocks.

[6] Geng A.L., Xu S.F., Zhang Y., Zhang J., Chu Q., Liu H.G. (2014). Effects of photoperiod on broodiness, egg-laying and endocrine responses in native laying hens. Br. Poult. Sci..

[7] Farghly M.F.A., Makled M.N. (2015). Application of Intermittent Feeding and Flash Lighting Regimens in Broiler Chickens Management. Egypt. J. Nutr. Feed..

[8] Kermanshahi H., Hadavi A. (2006). Effect of Added Extra Calcium Carbonate into the Diets, One Hour Before Starting Dark Period on Performance and Egg Quality of Laying Hens. Int. J. Poult. Sci..

[9] Roberts J.R. (2004). Factors Affecting Egg Internal Quality and Egg Shell Quality in Laying Hens. J. Poult. Sci..

[10] Saunders-Blades J.L., MacIsaac J.L., Korver D.R., Anderson D.M. (2009). The effect of calcium source and particle size on the production performance and bone quality of laying hens. Poult. Sci..

[11] Araujo J.A., Silva J.H., Costa F.G., Sousa J.M., Givisiez P.E., Sakomura N.K. (2011). Effect of the levels of calcium and particle size of limestone on laying hens. Rev. Bras. Zootec..

[12] Hrabia A., Scanes C.G., Dridi S. (2022). Chapter 35—Reproduction in the female. Sturkie’s Avian Physiology.

[13] Sinclair-Black M., Garcia R.A., Ellestad L.E. (2023). Physiological regulation of calcium and phosphorus utilization in laying hens. Front. Physiol..

[14] Korver D.R. (2020). Calcium nutrition, bone metabolism, and eggshell quality in longer-persisting layer flocks. Proc. Aust. Poult. Sci. Symp..

[15] Whitehead C.C., Fleming R.H. (2000). Osteoporosis in Cage Layers. Poult. Sci..

[16] Whitehead C.C. (2004). Overview of bone biology in the egg-laying hen. Poult. Sci..

[17] Aguado E., Pascaretti-Grizon F., Goyenvalle E., Audran M., Chappard D. (2015). Bone Mass and Bone Quality Are Altered by Hypoactivity in the Chicken. PLoS ONE.

[18] Garcia-Mejia R.A., Sinclair-Black M., Blair L.R., Angel R., Jaramillo B., Regmi P., Neupane N., Proszkowiec-Weglarz M., Arbe X., Cavero D. (2024). Physiological changes in the regulation of calcium and phosphorus utilization that occur after the onset of egg production in commercial laying hens. Front. Physiol..

[19] Nasr M.A.F., Murrell J., Wilkins L.J., Nicol C.J. (2012). Effect of keel fractures on egg-production parameters, mobility and behavior in individual laying hens. Anim. Welf..

[20] Whitehead C.C. (2004). Skeletal disorders in laying hens: The problem of osteoporosis and bone fractures. Welfare of the Laying Hen, Proceedings of the 27th Poultry Science Symposium of the World’s Poultry Science Association (UK Branch), Bristol, UK, 6 July 2003.

[21] Dunn I.C., Joseph N.T., Bain M., Edmond A., Wilson P.W., Milona P., Nys Y., Gautron J., Schmutz M., Preisinger R. (2009). Polymorphisms in eggshell organic matrix genes are associated with eggshell quality measurements in pedigree Rhode Island Red hens. Anim. Genet..

[22] Nys Y. (2017). Laying hen nutrition: Optimising hen performance and health, bone, and eggshell quality. Achiev. Sustain. Prod. Eggs.

[23] Arpasova H., Halaj M., Halaj P. (2010). Eggshell quality and calcium utilization in feed of Hens in repeated laying cycles. Czech J. Anim. Sci..

[24] Hunton P. (2005). Research on eggshell structure and quality: An historical overview. Braz. J. Poult. Sci..

[25] Rodriguez-Navarro A., Kalin O., Nys Y., Garcia-Ruiz J.M. (2002). Influence of the microstructure on the shell strength of eggs laid by hens of different ages. Br. Poult. Sci..

[26] Roberts J.R., Chousalkar K. (2013). Egg quality and age of laying hens: Implications for product safety. Anim. Prod. Sci..

[27] Benavides-Reyes C., Folegatti E., Dominguez-Gasca N., Litta G., Sanchez-Rodriguez E., Rodriguez-Navarro A.B., Faruk M.U. (2021). Research Note: Changes in eggshell quality and microstructure related to hen age during a production cycle. Poult. Sci..

[28] Bain M.M., McDade K., Burchmore R., Law A., Wilson P.W., Schmutz M., Preisinger R., Dunn I.C. (2013). Enhancing the egg’s natural defence against bacterial penetration by increasing cuticle deposition. Anim. Genet..

[29] Scott M.L., Hull S.J., Mullenhoff P.A. (1971). The Calcium Requirements of Laying Hens and Effects of Dietary Oyster Shell Upon Egg Shell Quality. Poult. Sci..

[30] Newman S., Leeson S. (1997). Skeletal integrity in layers at the completion of egg production. Worlds Poult. Sci. J..

[31] Makled M.N., Charles O.W. (1987). Eggshell Quality as Influenced by Sodium Bicarbonate, Calcium Source, and Photoperiod1. Poult. Sci..

[32] Clark A.J., Harrison C., Bragg A.J., House G.M., Stephan A.B., Arguelles-Ramos M., Ali A. (2024). Effect of Interrupting the Daily Scotophase Period on Laying Hen Performance, Bone Health, Behavior, and Welfare; Part I: Bone Health. Poultry.

[33] Johnson A.M., Anderson G., Arguelles-Ramos M., Ali A.A. (2022). Effect of dietary essential oil of oregano on performance parameters, gastrointestinal traits, blood lipid profile, and antioxidant capacity of laying hens during the pullet phase. Front. Anim. Sci..

[34] Bates D., Mächler M., Bolker B., Walker S. (2015). Fitting Linear Mixed-Effects Models Using lme4. J. Stat. Soft..

[35] Hothorn T., Bretz F., Westfall P. (2008). Simultaneous inference in general parametric models. Biom. J..

[36] Dunn I.C. Long Life Layer; genetic and physiological limitations to extend the laying period. Proceedings of the 19th European Symposium on Poultry Nutrition.

[37] Lennards R.M., Roland D.A.S.R. (1981). The influence of time of dietary calcium intake on shell quality. Poult. Sci..

[38] Jansen S., Bues M., Baulain U., Habig C., Halle I., Petow S., Sharifi A.R., Weigend A., Wilkens M.R., Weigend S. (2020). Bone health or performance? Adaptation response of genetically divergent chicken layer lines to a nutritive calcium depletion. Animals.

[39] Metwally M.A., Farghly M.F.A., Sharaqa T.M. (2021). Effects of light regimens and vitamin d3 levels and their interactions on broilers growth performance and carcass traits. Egypt. J. Nutr. Feed..

[40] Shen L., Shi Z.X., Li B.M., Wang C.Y., Ma H. (2012). The effect of lighting programmes on egg production and quality of Beijing you-chicken. In Animal Production Technology. Proceedings of the International Conference of Agricultural Engineering-CIGR-AgEng 2012: Agriculture and Engineering for a Healthier Life.

[41] Morris T.R. (2004). Environmental control for layers. Worlds Poult. Sci. J..

[42] Bryant S.L. (1987). A case for dawn and dusk for housed livestock. Appl. Anim. Behav. Sci..

[43] Savory C.J. (1980). Diurnal feeding patterns in domestic fowls: A review. Appl. Anim. Ethol..

[44] Romero C., Arija I., Viveros A., Chamorro S. (2022). Productive Performance, Egg Quality and Yolk Lipid Oxidation in Laying Hens Fed Diets including Grape Pomace or Grape Extract. Animals.

[45] Farghly M. (2014). Improvement of productive and reproductive performance of Dandarawi chicken through flash light program. Egypt. J. Anim. Prod..

[46] Lewis P.D., Backhouse D., Gous R.M. (2004). Constant photoperiods and sexual maturity in broiler breeder pullets. Br. Poult. Sci..

[47] Lewis P.D., Gous R.M. (2006). Constant and changing photoperiods in the laying period for broiler breeders allowed [corrected] normal or accelerated growth during the rearing period. Poult. Sci..

[48] Lewis P.D., Gous R.M. (2006). Effect of final photoperiod and twenty-week body weight on sexual maturity and early egg production in broiler breeders. Poult. Sci..

[49] Zhu H., Shao X., Chen Z., Wei C., Lei M., Ying S., Yu J., Shi Z. (2017). Induction of out-of-season egg laying by artificial photoperiod in Yangzhou geese and the associated endocrine and molecular regulation mechanisms. Anim. Reprod. Sci..

[50] Lewis P.D., Gous R.M., Ghebremariam W.K., Sharp P.J. (2007). Broiler breeders do not respond positively to photoperiodic increments given during the laying period. Br. Poult. Sci..

[51] Lewis P.D., Danisman R., Gous R.M. (2010). Photoperiods for broiler breeder females during the laying period. Poult. Sci..

[52] Geng A.L., Zhang Y., Zhang J., Wang H.H., Chu Q., Liu H.G. (2018). Effects of lighting pattern and photoperiod on egg production and egg quality of a native chicken under free-range condition. Poult. Sci..

[53] Farghly M.F., Mahrose K.M., Rehman Z.U., Yu S., Abdelfattah M.G., El-Garhy O.H. (2019). Intermittent lighting regime as a tool to enhance egg production and eggshell thickness in Rhode Island Red laying hens. Poult. Sci..

[54] Curtis P.A., Kerth L.K., Anderson K.E. (2005). Impact of strain on egg quality and composition during a single production cycle. Poult. Sci..

[55] Yuri F.M., Souza C.D., Schneider A.F., Gewehr C.E. (2016). Intermittent lighting programs for layers with different photophases in the beginning of the laying phase. Ciência Rural..

[56] Dikmen B.Y., Ipek A., Şahan Ü., Sözcü A., Baycan S.C. (2017). Impact of different housing systems and age of layers on egg quality characteristics. Turk. J. Vet. Anim. Sci..

[57] Nys Y., Gautron J. (2007). Structure and formation of the eggshell. Bioactive Egg Compounds.

[58] Hincke M.T., Nys Y., Gautron J., Mann K., Rodriguez-Navarro A.B., McKee M.D. (2012). The eggshell: Structure, composition and mineralization. Front. Biosci..

[59] Bell D. (1998). Egg Shell Quality: Its Impact on Production, Processing and Marketing Economics.

[60] Leeson S., Walker J.P., Summers J.D. (1982). Performance of laying hens subjected to intermittent lighting initiated at 24 weeks of age. Poult. Sci..

